# Bisphenol a increases risk for presumed non-alcoholic fatty liver disease in Hispanic adolescents in NHANES 2003–2010

**DOI:** 10.1186/s12940-018-0356-3

**Published:** 2018-02-01

**Authors:** Sofia G. Verstraete, Janet M. Wojcicki, Emily R. Perito, Philip Rosenthal

**Affiliations:** Division of Pediatric Gastroenterology, Hepatology, and Nutrition; Department of Pediatrics, Benioff Children’s Hospital San Francisco University of California San Francisco, Box 0136, 550 16th Street 5th Floor, San Francisco, CA 94143 USA

**Keywords:** NAFLD, BPA, Insulin resistance, Obesity, National Health and Nutrition Examination Survey

## Abstract

**Background:**

Bisphenol-A (BPA) is a ubiquitous chemical and recognized endocrine disruptor associated with obesity and related disorders. We explored the association between BPA levels and suspected non-alcoholic fatty liver disease (NAFLD).

**Methods:**

Unweighted analyses were used to study the relationship between urinary BPA levels and suspected NAFLD (alanine aminotransferase (ALT).

> 30 U/L, body mass index (BMI) Z-score > 1.064 and evidence of insulin resistance) using National Health and Nutrition Examination Survey (NHANES) data (2003–2010) on 12–19 year olds. Unweighted and weighted analyses were used to evaluate the risk with only elevated ALT.

**Results:**

We included 944 adolescents with urinary BPA and fasting laboratory tests from a total of 7168 adolescents. Risk of suspected NAFLD was increased in the second quartile of BPA levels (1.4–2.7 ng/mL) when compared to the first (< 1.4 ng/mL) (Odds Ratio (OR) 4.23, 95% Confidence Interval (CI) 1.44–12.41). The ORs for the third and second quartiles were positive but did not reach statistical significance. The association was stronger in Hispanics (*n* = 344) with BPA levels in the second (OR 6.12, 95% C.I. 1.62–23.15) quartile and when limiting the analyses to overweight/obese adolescents (*n* = 332), in the second (OR 5.56, 95% C.I. 1.28–24.06) and fourth BPA quartiles (OR 6.85, 95% C.I. 1.02–46.22) compared to the first quartile. BPA levels were not associated with ALT elevation.

**Conclusions:**

The risk of suspected NAFLD is increased in participants in higher quartiles of BPA exposure, particularly in those of Hispanic ethnicity. Further studies are required to fully understand the potential role of BPA in non-alcoholic fatty liver disease.

**Electronic supplementary material:**

The online version of this article (10.1186/s12940-018-0356-3) contains supplementary material, which is available to authorized users.

## Background

Non-alcoholic fatty liver disease (NAFLD) is the most common cause of chronic liver disease in children and adolescents in the United States [1–4] with up to 38% of obese children affected [5]. Prevalence increases with age, reaching approximately 17.5% in adolescents [5], and with 5 times the risk of fatty liver in Hispanics [5–7]. Although it is a histologic diagnosis, NAFLD is clinically suspected when steatosis is identified in imaging studies or with elevation of transaminases in otherwise healthy children, particularly in overweight children or those with evidence of insulin resistance [8]. While genetics clearly play some role in NAFLD [9, 10], environmental exposures may also be inciting or exacerbating factors [11, 12].

Bisphenol-A (BPA) is a ubiquitous chemical and likely endocrine disruptor [13]. It was first synthesized in the 1940s as a synthetic estrogen and is now used in thermal receipt paper as well as coatings that prevent metal corrosion of food and beverage containers [14]. In vitro and animal studies have shown that BPA contributes to de novo fatty acid synthesis [15], stimulates the accumulation of triacylglycerol in adipocytes and human hepatocellular carcinoma cells [16] and up- regulates gene expression involved in lipid metabolism and insulin resistance (IR) [17, 18]—all mechanisms that may contribute to NAFLD. In humans, obesity and diabetes have been associated with serum and urine concentrations of some environmental chemicals, leading to the hypothesis that these chemicals interfere with aspects of metabolism [19–21]. In adults, exposure to BPA is associated with obesity, type 2 diabetes mellitus (DM), cardiovascular disease, and increased serum gamma glutamyl transpeptidase and alkaline phosphatase levels [22, 23]. Dose responsive associations have been reported between urinary BPA and fasting blood glucose and metabolites of oxidative stress [24, 25], which also contribute to the development of NAFLD. Although previous cross-sectional studies have found that urinary BPA concentration was significantly associated with obesity in children and adolescents [26–28], the association between elevated BPA and NAFLD has not been studied.

The aim of this study is to specifically explore the relationship between BPA exposure in US adolescents and suspected NAFLD defined as elevated ALT levels, overweight or obesity and evidence of insulin resistance. Secondary aims include stratification of the above relationship according to race/ethnicity given the high prevalence of NAFLD in Hispanic adolescents, and assessing the relationship between BPA exposure in US adolescents and ALT elevation.

## Methods

### Study design

The National Health and Nutrition Examination Survey (NHANES) [29] is a nationally representative survey of the civilian non-institutionalized U.S. population carried out by the National Center for Health Statistics (NCHS) of the Centers for Disease Control and Prevention (CDC). Survey cycles from 2003–2004 to 2009–2010 were used to obtain the questionnaire, laboratory, diet and physical examination components for our analysis. Previous cycles that did not include urinary BPA levels and posterior cycles where fasting laboratory levels had not been published at the time of the analysis were excluded. NHANES is approved by the NCHS Research Ethics Review Board, and written informed consent and child assent (as appropriate) were obtained from participants. The Institutional Review Board at the University of California, San Francisco (Committee for Human Research) exempted the present study from review.

### Inclusion criteria

**Fig. 1:**
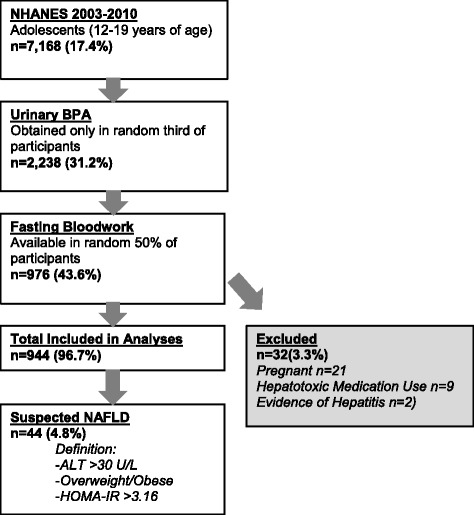
Population Included in Analysis

Adolescent participants between the ages of 12 and 19 were selected for inclusion in this study. Urinary BPA levels were available for a random third and fasting laboratory results were available for a separate random half of participants (both randomly selected by NHANES [30]). Only those included in both random subsets were included in our overall analysis. Participants who were pregnant or known to have Hepatitis B or C, Human Immunodeficiency Virus (HIV), known liver disease, or known exposure to hepatotoxic medication (see Supplementary Table 1) were excluded from the analysis. The inclusion process is detailed in Fig. 1.

### Method for BPA concentration assessment

A randomly selected subsample had total urinary BPA concentration measured in a urine sample for each participant [31, 32]. Concentrations were obtained using high performance liquid chromatography and tandem mass spectroscopy [30]. A value of 0.28 ng/mL was substituted when urinary BPA concentrations were below the level of detection, as is routinely assigned by the NHANES [33]. One hundred patients (3.5%) had urinary concentrations below this level. Urinary creatinine was included in all multivariate models to correct for urinary dilution, as done in previous publications [34, 35]. Our primary predictor was BPA level categorized into quartiles. Log-transformed total concentrations of BPA levels were also estimated and used independently as a predictor in the analyses.

### Definition of suspected non-alcoholic fatty liver disease (NAFLD)

Our primary study outcome was the presence or absence of suspected NAFLD. Participants were determined to have suspected NAFLD if they had elevated alanine transaminase (ALT) (ALT ≥30 U/L) in combination with overweight [BMI *z* score of 1.036 or greater (85th percentile for age and sex)] or obesity [BMI z- score 1.064 or greater (95th percentile for age and sex)] and evidence of insulin resistance [Homeostatic Model Assessment for Insulin Resistance (HOMA-IR) ≥3.16]. HOMA-IR was calculated by dividing the product of fasting insulin (mU/L) times fasting plasma glucose (mmol/L) by 22.5 [36]. BMI z-scores were derived using the norms suggested by the Centers for Disease Control and Prevention [37]. Secondary analyses were conducted using elevated ALT (ALT ≥30 U/L) [38] as an outcome. Sensitivity analyses were conducted using a higher cutoff for ALT (> 50 U/L in boys, > 44 U/L in girls) as a more specific marker for identifying obese children at high risk for NAFLD [39].

### Definition and inclusion of potential confounders

Potential confounders were identified in the current literature [26–28, 40]. Statistical modeling was used to determine causal relationships. Variables that were identified as possible confounders and were available in NHANES included age, gender, race/ethnicity, country of birth, poverty to income ratio, survey cycle year exposure to tobacco using serum cotinine as a surrogate, and total caloric intake in 24 h. Race/ethnicity were categorized based on self-report by caregivers in participants under 16 years of age, and by participants in those over 17 years of age. Given that exposure to tobacco smoke is a risk factor for metabolic syndrome in adolescence, serum cotinine level was included in all multivariate models as a continuous variable. A variable characterizing survey cycle year was included in all multivariable models since median urinary BPA levels were not uniform across survey cycles. Urinary BPA in the 2003–2004 cycle had a median of 4.3 ng/mL (IQR 2–7.5), while levels for the 2005–2006, 2007–2008, and 2009–2010 cycles were 2.4 ng/mL, 2 ng/mL, and 2.1 ng/mL respectively.

### Statistical analysis

Multivariate logistic regression models were conducted using Stata 12.0 (Stata Corp, College Station TX) following NCHS recommendations. Following the practice of Trasande [41] and Stahlhut [42], unweighted analyses were used as our primary analytic approach since the studied population consisted of two overlapping NHANES subsamples. Although there are subsample weights for each, combining these would decrease the sample size ultimately resulting in unstable and unreliable statistical estimates. Additional sensitivity analyses were performed. For the secondary analysis of ALT elevation, we performed both an unweighted analysis for comparison with our primary outcome, and a weighted analysis following the NCHS guidelines to adjust for the complex survey structure of NHANES. All multivariate models were adjusted for urinary creatinine concentrations to account for dilution of urinary BPA.

## Results

In the four NHANES cycles studied (2003–2010), 7168 adolescents between the ages of 12 and 19 were evaluated. Urinary BPA concentrations were obtained in 2238 (31.2%). Fasting laboratory values needed to compute HOMA-IR (insulin and glucose) were available in a second randomized subsample of 976 participants (43.6%). Thirty-two participants (3.3%) were excluded due to pregnancy (*n* = 21), exposure to hepatotoxic medications (*n* = 9), or evidence of hepatitis B or C (*n* = 2). None had HIV or other known liver disease. Our primary, unweighted analysis thus included 944 adolescents from 12 to 19 years of age with both fasting bloodwork and urinary BPA concentrations. The population selected is illustrated in Fig. 1.

For the analysis of ALT elevation, 2105 participants had available BPA concentrations and ALT. Of these, 1978 participants were included in secondary weighted and unweighted analyses after exclusions for pregnancy (*n* = 38), hepatitis B or C (*n* = 5), and exposure to hepatotoxic medications (*n* = 30).

Descriptive and bivariate analyses of urinary BPA quartiles and demographic, environmental, and dietary data for those included in the study (*n* = 944) are presented in Table 1. Median urinary BPA in the suspected NAFLD cohort was 2.6 ng/mL (interquartile range, IQR, 1.3–5.3 ng/mL). ALT was elevated in 8.4% *n* = 80), and suspected NAFLD, as defined above, was present in 4.8% (*n* = 44) (Table 1). For the secondary analysis (*n* = 1978) including the larger sample of those with ALT and BPA concentrations only, median urinary BPA was 4.73 (IQR 0.28–149) and ALT was elevated in 9.81% (*n* = 194) (results not shown).

**Table 1:** Demographic data by Bisphenol-A Quartile for NHANES Population^g^

	n (%)	Bisphenol-A quartile				
		0.28–1.3 ng/mL	1.4–2.7 ng/mL	2.8–5.4 ng/mL	≥5.5 ng/mL	*p-*value
	*n* = 944	*n* = 250 (%)	*n* = 240 (%)	*n* = 225 (%)	*n* = 228	
Age (years)^f^	15.5 ± 2.3	15.1 ± 2.2	15.5 ± 2.3	15.6 ± 2.3	15.6 ± 2.4	0.03
Gender (male)	523 (55.4)	141 (56.4)	134 (55.8)	118 (52.4)	130 (56.8)	0.78
Country of Birth						
United States	822 (87.1)	213 (85.2)	207 (86.3)	204 (90.7)	198 (86.5)	
Mexico	69 (7.3)	19 (7.6)	21 (8.8)	13 (5.8)	16 (7.0)	0.51
Other	53 (5.6)	18 (7.2)	12 (5.0)	8 (3.6)	15 (6.6)	
Poverty to Income Ratio^f^	2.07 + 1.51	2.11 ± 1.48	2.10 ± 1.49	2.00 ± 1.51	2.08 ± 1.56	0.87
Race/Ethnicity						
Non-Hispanic White	256 (27.1)	61 (24.4)	66 (27.5)	58 (25.8)	71 (31)	
Hispanic	344 (36.4)	117 (46.8)	98 (40.8)	74 (32.9)	55 (24)	< 0.001
Non-Hispanic Black	297 (31.5)	54 (21.6)	67 (27.9)	82 (36.4)	94 (41.1)	
Other	47 (5.0)	18 (7.2)	9 (3.8)	11 (4.9)	9 (3.9)	
Tobacco Exposure (by cotininelevel)						
Low (< 0.015 ng/mL^a^)	168 (18.0)	59 (23.7)	52 (21.8)	38 (17.1)	19 (8.4)	
Medium (0.015 to < 2 ng/mL)	603 (64.4)	159 (63.9)	150 (62.8)	140 (63.1)	154 (68.1)	< 0.001
High (≥2 ng/mL)	165 (17.6)	31 (12.5)	37 (15.5)	44 (19.8)	53 (23.5)	
Daily Caloric Intake (kilocalories)^f^	2270.9 ± 1030.3	2319.8 ± 1090.3	2347.8 ± 1069.8	2209.1 ± 940.0	2199.6 ± 1005.9	0.30
BMI Z-Score ≥ 1.036 (≥85th Percentile)	332 (35.6)	80 (32.3)	87 (36.9)	79 (3.3)	86 (38.4)	0.54
Metabolic Syndrome	101 (10.8)	29 (11.7)	23 (9.7)	22 (9.9)	27 (12.0)	0.80
Hypertriglyceridemia^b^	97 (10.3)	23 (9.2)	23 (9.6)	23 (10.3)	28 (12.3)	0.70
Low High Density Lipoprotein^c^	249 (26.6)	71 (28.5)	51 (21.3)	64 (28.8)	63 (28.0)	0.20
Hypertension^d^	260 (27.6)	62 (24.8)	61 (25.4)	62 (27.6)	75 (32.8)	0.20
Waist Circumference > 90th	201 (21.8)	51 (20.6)	51 (21.9)	48 (21.8)	51 (23.1)	0.93
Percentile						
Impaired fasting glucose	189 (20)	59 (23.6)	57 (23.8)	36 (16.0)	37 (16.2)	0.04
Alanine Aminotransferase > 30 U/L^e^	80 (8.58)	18 (7.23)	29 (12.18)	14 (6.33)	19 (8.48)	0.11
Suspected Non-Alcoholic Fatty Liver Disease	44 (4.8)	7 (2.83)	17 (7.3)	9 (4.1)	11 (5.1)	0.14

^a^To convert cotinine to nmol/L multiply by 5.675, ^b^Serum triglycerides ≥150 mg/dL (for conversion to mmol/L multiply by 0.0113), ^c^HDL < 40 mg/dL (for conversion to mmol/L multiply by 0.259), ^d^Systolic and/or diastolic blood pressure > 85th percentile for age/height, ^e^To convert to ukat/L multiply by 0.0167, ^f^Mean ± SD, ^g^NHANES population limited to those with fasting lab draws and body mass index measurements in addition to BPA

For the smaller cohort with fasting labs and weights, BPA concentrations in Hispanic participants were lowest (median BPA 2.1 ng/mL, IQR 1–3.8), with slightly higher concentrations in non-Hispanic white participants (median BPA 2.8 ng/mL, IQR 1.4–6) and significantly higher concentrations in non-Hispanic black participants (median BPA 3.6 ng/mL, IQR 1.7–6.5) (results not shown). This difference in BPA concentrations among race/ethnicity categories is also reflected in the distribution of race/ethnicity among quartiles of urinary BPA (*p* < 0.001; see Table 1).

### Urinary BPA and non-alcoholic Steatohepatitis

In logistic regression models adjusting only for urinary creatinine, there were no no associations between suspected NAFLD and increasing BPA concentrations (Table 2). In multivariate models adjusting for all confounders, the odds of suspected NAFLD were increased in the second quartile of urinary BPA concentration [Odds Ratio (OR) 4.23, 95% Confidence Interval (CI) 1.44–12.41] (Table 2). The association was not observed in the third and fourth quartiles of BPA concentrations.]

**Table 2:** Risk of Suspected Non-Alcoholic Fatty Liver Disease (NAFLD)

Risk of Suspected NAFLD^a^ in total population								
	All races (*n* = 944)				Non-hispanic white (*n* = 256)		Hispanic (*n* = 344)	
	Unadjusted model^d^		Adjusted model^e^		Adjusted model^f^		Adjusted model^f^	
	OR (95% C.I.)^c^	*p-*value	OR (95% C.I.)	*p-*value	OR (95% C.I.)	*p-*value	OR (95% C.I.)	*p*-value
Urinary BPA^b^ Category								
0.28–1.3 ng/mL	Reference		Reference		Reference		Reference	
1.4–2.7 ng/mL	2.66 (0.94–7.48)	0.064	4.23 (1.44–12.41)	0.010	1.69 (0.26–10.92)	0.571	6.12 (1.62–23.15)	0.009
2.8–5.4 ng/mL	1.95 (0.57–6.71)	0.281	2.43 (0.57–10.43)	0.225	1.19 (0.09–15.63)	0.887	2.74 (0.42–17.70)	0.281
≥5.5 ng/mL	2.12 (0.58–7.72)	0.248	3.39 (0.78–14.80)	0.101	1.30 (0.06–27.62)	0.862	4.21 (0.74–24.11)	0.104
Log Transformed BPA								
	1.07 (0.69–1.67)	0.757	1.20 (0.75–1.91)	0.444	1.22 (0.48–3.12)	0.674	1.02 (0.61–1.72)	0.937

^a^Non-Alcoholic Fatty Liver Disease, ^b^Bisphenol A, ^c^Odds Ratio (95% Confidence Interval), ^d^Adjusted for urinary creatinine, ^e^Adjusted for urinary creatinine, gender, age, race, family income to poverty ratio, country of birth, total caloric intake in 24 h, cotinine levels, and survey cycle, ^f^Adjusted for urinary creatinine, gender, age, total caloric intake in 24 h, cotinine levels, and survey cycle

Suspected NAFLD was present in 24 (7.04%) Hispanic, 12 (4.88%) non-Hispanic white, 6 (2.09%) non-Hispanic black, and 2 (4.55%) other race participants out of the 44 total (result not shown). Multivariate analysis in non-Hispanic White and Hispanic populations showed that Hispanic participants with urinary BPA in the second quartile of BPA concentrations were at higher risk for suspected NAFLD than those in the lowest quartile of BPA (Table 2). Suspected NAFLD was too rare in non-Hispanic black and other categories to evaluate the role of BPA quartile on suspected NAFLD in stratified analyses.

The analyses were repeated including only overweight and obese participants (Table 3). There was a stronger association between suspected NAFLD and the second quartile of BPA concentrations (OR 5.56, 95% C.I. 1.28–24.06) (Table 3). When limiting the analysis to overweight and obese Hispanic participants, there was an even higher risk of suspected NAFLD in the second BPA quartile (OR 5.00, 1.40–17.93; *p* = 0.015) (Table 3).

**Table 3:** Risk of Suspected Non-Alcoholic Fatty Liver Disease (NAFLD) in Overweight/Obese Adolescents

Risk of Suspected NAFLD^a^ in overweight/obese participants (BMI^b^ Z-Score ≥ 1.064)								
	All races (*n* = 327)				Non-hispanic white (*n*=)		Hispanic (*n* = 126)	
	Unadjusted model^d^		Adjusted model^e^		Adjusted model^f^		Adjusted model^f^	
	OR (95% C.I.)^c^	*p-*value	OR (95% C.I.)	*p-*value	OR (95% C.I.)	*p-*value	OR (95% C.I.)	*p-*value
Urinary BPA^g^ Category								
0.28–1.3 ng/mL	Reference		Reference				Reference	
1.4–2.7 ng/mL	2.86 (0.98–8.29)	0.053	5.56 (1.28–24.06)	0.023	1.29 (0.12–13.89)	0.829	5.00 (1.40–17.93)	0.015
2.8–5.4 ng/mL	2.20 (0.61–7.93)	0.224	3.39 (0.60–19.13)	0.162	1.42 (0.04–45.42)	0.841	1.98 (0.23–17.43)	0.527
≥5.5 ng/mL	2.31 (0.66–8.01)	0.181	6.85 (1.02–46.22)	0.048	0.87 (0.05–16.55)	0.926	5.20 (0.93–29.01)	0.060
Log Transformed BPA								
	1.11 (0.71–1.73)	0.644	1.41 (0.74–2.70)	0.285	1.28 (0.19–8.59)	0.787	1.04 (0.58–1.85)	0.896

^a^Non-Alcoholic Fatty Liver Disease, ^b^Body Mass Index, ^c^Odds Ratio (95% Confidence Interval), ^d^Adjusted for urinary creatinine, ^e^Adjusted for urinary creatinine, gender, age, race, family income to poverty ratio, country of birth, total caloric intake in 24 h, cotinine levels, and survey cycle, ^f^Adjusted for urinary creatinine, gender, age, total caloric intake in 24 h, cotinine levels, and survey cycle, ^g^Bisphenol A

### Urinary BPA and elevated ALT

In our secondary analysis of weighted analyses elevated ALT alone, there were no significant associations with urinary BPA in the second (OR 1.34, 95% C.I. 0.57–3.11), third (OR 1.13, 95% C.I. 0.69–2.66), or fourth (OR 1.35, 95% C.I. 0.57–3.20) quartiles when compared to the first quartile in multivariate models. Additionally, no associations were found in univariate models, when evaluating BPA as a log-transformed continuous variable, after stratification for race/ethnicty or in any unweighted analyses using our standard cut-off limit for ALT (> 30 U/L) or after sensitivity analyses using higher cutoffs (data not shown).

## Discussion

This is the first study to evaluate the association between BPA and suspected NAFLD. In a relatively large and well-characterized sample of US adolescents we found that the odds of suspected NAFLD were higher in the second quartile of BPA concentrations. Additionally, this association was higher in Hispanic adolescents. These associations persisted  after limiting analyses to only the Hispanic population. ALT elevation was not associated with BPA concentration.

We hypothesize that we had an improved ability to detect an association in Hispanic adolescents because the prevalence of NAFLD in Hispanic participants (7%) was higher than in non-Hispanic whites (4.8%), non-Hispanic blacks (2.1%) or other participants (4.6%) in our population. We suspect that low prevalence of elevated ALT and suspected NAFLD in the cohort may have made it harder to detect an association in the higher BPA quartiles. Furthermore, wide confidence intervals in these quartiles suggest that a small sample size hindered detection of an association between urinary BPA and suspected NAFLD in the upper BPA quartiles. Alternatively, a non-monotonic dose-response relationship, where the risk of disease is highest with low, constant doses, may exist with exposure to BPA. In animal studies, metabolic dysfunction has been identified with exposure to “low doses” (doses generally considered safe for human exposure) of BPA [25].

One prior publication explored the relationship between BPA and elevated serum transaminases in boys. In a small group (*n* = 39) of overweight and obese 3 to 8 year olds, elevated serum aspartate aminotransferase (AST) was associated with increasing BPA concentrations [43] in male participants. In a population based adult study, serum gamma-glutamyl transpeptidase elevation was also associated with elevated BPA concentrations [22]. Neither of these studies excluded other causes of liver disease.

We focused our definition of suspected NAFLD with the aim of increasing the specificity of the diagnosis by excluding other causes of elevated ALT concentrations. The association between BPA and suspected NAFLD was positive in using the more specific definition including obesity and insulin resistance, but consistently negative when using elevated ALT alone as an outcome, which strengthens our hypothesis that BPA is associated with NAFLD.

Obesity has been associated with BPA in previous studies [26–28, 44], independent of any liver disease, which raised the concern that the association with suspected NAFLD may be due to using BMI ≥85th percentile as part of the definition for suspected NAFLD. Limiting the analysis to overweight or obese participants allowed us to assess the strength of the association between BPA and NAFLD independent of weight categories. We found stronger associations between BPA and NAFLD among the overweight and obese than among the total cohort of 944. This suggests that BPA may be contributing to the development of NAFLD, particularly in Hispanics who are overweight/obese. Further studies should investigate a possible interaction between BPA consumption and higher weight categories and possible development of NAFLD.

This study’s limitations are similar to those of other studies of BPA and obesity or related outcomes. The bulk of the literature studying these associations consists of cross-sectional data [26–28, 40, 43–45], with the largest studied groups coming from NHANES [26, 28, 43]. All of the studies evaluating these associations use urinary concentrations of BPA to determine exposure. Urinary BPA concentrations are obtained from a single sample, which is thought to reflect recent (4-40 h) dietary intake and does not necessarily reflect chronically high (or low) concentrations [32, 46] or long term exposure. Moreover, studies have shown high variability within individuals [47], as well as detectable concentrations after fasting [48, 49], which suggest urinary BPA concentrations may also reflect non-dietary exposure or accumulation in tissue. Specifically, studies have shown that BPA has a strong affinity for adipose tissue with accumulation three times higher than in other tissues [49]. BPA is ubiquitous in the environment but it is difficult to evaluate long-term exposure with current techniques.

BPA and NAFLD both inhibit human hepatic metabolic activities [50, 51]. Additionally, BPA is subject to hepatic metabolism where it is converted to an inactive monoglucuronide form [50]. This is another important limitation to our study- BPA metabolism may be impaired in NAFLD due to defective hepatic glucuronidation increasing total BPA levels in urine. Studies in animal models, patients affected by liver disease of other etiologies and longitudinal studies in humans etiologies are necessary to clarify this conundrum.

The relationship between BPA and NAFLD identified in this large, nationally representative sample of adolescents should encourage further research on the effect of BPA and obesity related outcomes, particularly adequately sized longitudinal studies to study causality. Further longitudinal studies would help elucidate whether BPA concentrations are a risk factor for NAFLD or are increased in obese children because they have greater adipose stores of this lipophilic chemical. The association we found with NAFLD suggests that BPA concentrations may be tied to end-organ disease, not just co-existent with obesity.

## Conclusion

In adolescents, particularly those of Hispanic ethnicity, there is an association between Bisphenol-A and suspected non-alcoholic fatty liver disease. Given the cross-sectional nature of this study, we are unable to establish true causality. Hispanics may be more prone to eating poor quality foodstuff with additional environmental toxins in addition to BPA which may have a cumulative impact on risk for NAFLD. Our study further indicated that there may important interactive effects between overweight, obesity and ingestion of BPA and development of NAFLD. It is possible that BPA accumulates in adipose tissues as other studies have indicated. Further longitudinal studies evaluating long-term exposure to BPA, are needed to fully understand the role of BPA and other environmental toxins in NAFLD, as well as possible mechanisms of injury.

## Additional file

Additional file 1: Table S1. List of Hepatotoxic Medications (DOCX 95 kb)

## Abbreviations

ALT: Alanine amino transferase
AST: Aspartate amino transferase
BPA: Bisphenol-A
CDC: Centers for disease control
CI: Confidence interval
DM: Diabetes mellitus
NAFLD: Non-alcoholic fatty liver disease
NASH: Non-alcoholic steato-hepatitis
NCHS: National Center for Health Statistics
NHANES: National Health and Nutrition E*** S***
OR: Odds Ratio
