# Supplementary material for: Bisphenol a increases risk for presumed non-alcoholic fatty liver disease in Hispanic adolescents in NHANES 2003–2010
Source: Environ Health. 2018 Feb 1;17:12. doi: 10.1186/s12940-018-0356-3 (PMC5796302; doi:10.1186/s12940-018-0356-3)
Supplement: Additional file 1: Table S1. — List of Hepatotoxic Medications (DOCX 95 kb) [file 12940_2018_356_MOESM1_ESM.docx]

**Supplementary Table 1: List of Hepatotoxic Medications**

| **Hepatotoxic Medications** | |
| --- | --- |
| ABACAVIR SULFATE | MESALAMINE |
| ACETAMINOPHEN | MESTRANOL |
| ALLOPURINOL | NORETHINDRONE |
| AMIODARONE HYDROCHLORIDE | METHIMAZOLE |
| AMOXICILLIN | METHOTREXATE SODIUM |
| AMOXICILLIN TRIHYDRATE | METHYLDOPA |
| ATORVASTATIN CALCIUM | MINOCYCLINE HYDROCHLORIDE |
| AZATHIOPRINE | MONTELUKAST SODIUM |
| BICALUTAMIDE | NABUMETONE |
| BROMOCRIPTINE MESYLATE | NIACIN |
| CARBAMAZEPINE | NITROFURANTOIN |
| CARBIDOPA | NORETHINDRONE |
| LEVODOPA | OLANZAPINE |
| CERIVASTATIN SODIUM | PEMOLINE |
| CHLORPROPAMIDE | PERMETHRIN |
| CHLORZOXAZONE | PHENAZOPYRIDINE HYDROCHLORIDE |
| CLAVULANATE POTASSIUM | PHENYTOIN SODIUM |
| CLOTRIMAZOLE | PILOCARPINE HYDROCHLORIDE |
| COLCHICINE | PRAVASTATIN SODIUM |
| DESOGESTREL | PROBENECID |
| ETHINYL ESTRADIOL | PROGESTERONE |
| DICLOFENAC | PROPYLTHIOURACIL |
| DIDANOSINE | PYRAZINAMIDE |
| DIVALPROEX SODIUM | QUININE SULFATE |
| EFAVIRENZ | REPAGLINIDE |
| ERYTHROMYCIN | RIFABUTIN |
| ESTROGEN | RITONAVIR |
| ETHAMBUTOL HYDROCHLORIDE | RIVASTIGMINE TARTRATE |
| FENOFIBRATE | SAQUINAVIR |
| FLUCONAZOLE | SIMVASTATIN |
| FLUOROURACIL | STAVUDINE |
| FLUTAMIDE | SULFAMETHOXAZOLE |
| FLUVASTATIN SODIUM | SULFASALAZINE |
| GLIMEPIRIDE | TAMOXIFEN CITRATE |
| GLIPIZIDE | TERBINAFINE HYDROCHLORIDE |
| GLYBURIDE | TESTOSTERONE |
| INDINAVIR SULFATE | TETRACYCLINE HYDROCHLORIDE |
| ISONIAZID | TIZANIDINE HYDROCHLORIDE |
| ITRACONAZOLE | TOLAZAMIDE |
| KETOCONAZOLE | TRETINOIN |
| LABETALOL HYDROCHLORIDE | TRIMETHOPRIM |
| LAMIVUDINE | TROGLITAZONE |
| LEFLUNOMIDE | VALPROIC ACID |
| LEUCOVORIN CALCIUM | VALPROATE |
| LOVASTATIN | ZAFIRLUKAST |
| MEDROXYPROGESTERONE ACETATE | ZIDOVUDINE |
| MEGESTROL ACETATE |  |
